# Early Sirolimus Gel Treatment May Diminish Angiofibromas and Prevent Angiofibroma Recurrence in Children With Tuberous Sclerosis Complex

**DOI:** 10.3389/fmed.2020.00001

**Published:** 2020-01-22

**Authors:** Tohru Okanishi, Ayataka Fujimoto, Hideo Enoki, Masaaki Ogai

**Affiliations:** ^1^Tuberous Sclerosis Complex Board, Seirei Hamamatsu General Hospital, Hamamatsu, Japan; ^2^Department of Child Neurology, Seirei Hamamatsu General Hospital, Hamamatsu, Japan; ^3^Comprehensive Epilepsy Center, Seirei Hamamatsu General Hospital, Hamamatsu, Japan; ^4^Department of Dermatology, Seirei Hamamatsu General Hospital, Hamamatsu, Japan

**Keywords:** angiofibroma, sirolimus, fibrous plaque, hypopigmented macules, tuberous sclerosis complex, early treatment

## Abstract

**Introduction:** Tuberous sclerosis complex (TSC) is a multisystem neurocutaneous disorder. Angiofibromas (AF), fibrous plaques, and hypopigmented macules are the major skin findings in TSC. Topical sirolimus reduces the volume and redness of AF and other skin findings. However, the efficacy of early intervention and long-term treatment remains to be clarified. We investigated the efficacy of sirolimus gel for AF in children with TSC.

**Methods:** We recruited nine children (five boys; four girls) with TSC and AF. We used 0.2% sirolimus gel over 6 months. We reviewed each patient's medical records and photographs for clinical information and data related to improvements in skin lesions. We evaluated the size of AF, fibrous plaques, and color changes in AF and hypopigmented macules.

**Results:** Age at the initiation of treatment ranged from 3.5 to 11.0 years. The follow-up period ranged from 6 to 36 months (≥24 months in 3 children). Patients presented with papular AF (9), miliary AF (8), AF redness (9), fibrous plaques (5), and hypopigmented macules (2). After 6 months of treatment, improvement of AF size and redness was seen in all nine patients. Patients treated for ≥24 months showed significant decrease in AF size that persisted until the final follow-up. Gradual improvement in fibrous plaques was observed, and marked reduction in size was achieved by 4–18 months.

**Conclusion:** Early sirolimus gel intervention is effective for the treatment of AF and fibrous plaques in children with TSC. Early intervention with sirolimus gel may maintain the skin at near-normal levels in patients with TSC.

## Introduction

Tuberous sclerosis complex (TSC) is a multisystemic neurocutaneous disorder caused by mutations in either the *TSC1* or *TSC2* gene. TSC is characterized by hamartomas in multiple organs including the skin, central nervous system, eyes, heart, lungs, and kidneys. TSC1 (hamartin) and TSC2 (tuberin) form a TSC protein complex that acts as an inhibitor of the mammalian target of rapamycin pathway, which regulates cell growth, cell proliferation, autophagy, and protein/lipid synthesis (1, 2). Vogt et al. proposed a diagnostic triad consisting of seizure, intellectual disability, and facial angiofibromas (AF) (3). This triad had been used for 60 years until Gomez suggested that TSC exists along a clinical spectrum (4). In 2012, the International Tuberous Sclerosis Complex Consensus Conference (5) developed revised diagnostic criteria for TSC, including 11 major and six minor features. The dermatological features outlined in these criteria include AF, fibrous cephalic plaques, hypopigmented macules, ungual fibromas, and shagreen patches (major) as well as Confetti skin lesions (minor) (5). Hypopigmented macules are seen at birth, while facial AF, fibrous cephalic plaques, and shagreen patches are observed beginning in early childhood. Ungual fibromas manifest during adolescence or adulthood (6–8). These often disfiguring skin lesions significantly impair patients' quality of life. Invasive therapeutic modalities such as surgical removal or laser therapy are often utilized but are limited by pain and potential for scarring.

Hofbauer et al. reported the first case of a patient with TSC who received systemic sirolimus as immunosuppressive treatment after kidney transplantation, following which AF improvement was observed (9). Recent studies have indicated that topical sirolimus gel/ointment is effective for the treatment of AF in patients with TSC (7, 8, 10–16). Wataya-Kaneda et al. conducted a randomized clinical trial to confirm the efficacy of sirolimus gel for AF in patients with TSC. In this trial, 18 of 30 patients (60%) treated with sirolimus gel showed improvement after 12 weeks, while no patients in the placebo group (*n* = 31) improved (8). Koenig et al. also conducted a randomized clinical trial that included 179 patients. Responder rates for 1 and 0.1% sirolimus were 82 and 66%, respectively (12). These studies demonstrated the remarkable efficacy of topical sirolimus for AF. However, it remains unclear if sirolimus gel treatment can diminish AF and keep the skin normal for TSC children with early stages of AF. Therefore, in the present study, we aimed to investigate whether early intervention with sirolimus gel can improve the facial skin lesions to the normal skin level and manage the condition for a long time.

## Materials and Methods

### Patients

We retrospectively recruited patients with TSC who had visited Seirei Hamamatsu General Hospital between March 2016 and June 2019 using the Tuberous Sclerosis Complex Board database. The patient inclusion criteria were as follows: (a) definitive diagnosis of TSC in accordance with modified Gomez criteria, (b) presence of facial AF, (c) sirolimus gel treatment for AF, (d) age under 15 at the beginning of the treatment, and (e) follow up for >6 months. Patients with (a) erosion or ulcer associated with AF, (b) poor adherence to treatment, and (c) history of laser therapy or surgery for AF were excluded.

We reviewed each patient's medical records for the following clinical information: sex, age at the beginning of the sirolimus gel treatment, treatment with oral everolimus or sirolimus, and follow-up period. We also reviewed data regarding AF, fibrous plaques, hypopigmented macules, and shagreen patches. The skin manifestations reviewed are described in section Evaluation of Skin Lesions and Treatment Efficacy.

### Sirolimus Gel Treatment for Skin Lesions

Each patient was instructed to evenly spread experimental or commercially supplied 0.2% sirolimus gel over all facial skin lesions including AF, fibrous plaques, and hypopigmented macules twice a day. Under the regulatory approved treatment criteria in Japan (https://nobelpark.jp/product/pdf/rapalimus_gel_pr.pdf [in Japanese]), the maximum daily dosages were 0.4 g (0.8 mg) for patients <6 years old, 0.6 g (1.2 mg) for patients between 6 and 11 years old, and 0.8 g (1.6 mg) for patients >11 years old. Sirolimus gel was applied by guardians for all nine patients.

### Evaluation of Skin Lesions and Treatment Efficacy

All patients were examined for AF, fibrous plaques, and hypopigmented macules at the beginning of treatment (baseline) and were followed up for at least 1, 2, 3, 4, and 6 months after treatment. At each visit, patients were medically examined, and their facial lesions were usually photographed with the same digital camera from the front and right/left sides of the face (45° angle).

At the beginning of the treatment, the severity of the lesions was evaluated according to the following items. For the estimations of severity about the miliary AF and color of AF, we referred to FASI score in the previous study (17):

1. Papular AF: number of papular AF with a diameter >2 mm.
2. Miliary AF: miliary grouped small AF with a diameter <2 mm and their extension (covering or not covering >50% of the cheek surface).
3. Color of AF: redness of AF.
4. Fibrous plaque: number of fibrous plaque.
5. Hypopigmented macules: number of hypopigmented macules.

At each visit, we evaluated treatment efficacy based on authors' opinions (TO or MO) and conversations with guardians. Lesions were evaluated according to the following items:

1. Size of papular AF: height of papular AF with a diameter >2 mm.
2. Miliary AF: changes in the smoothness of diffusely distributed, multiple miliary AF on the nose, cheeks, and forehead.
3. Color of AF: improvements in the redness of papular/miliary AF (<2 mm) when compared with the perilesional skin.
4. Size of fibrous plaques: height of fibrous plaques.
5. Color of hypopigmented macules: whiteness of the lesion when compared with the perilesional skin.

The authors (TO and MO) reviewed the baseline severity of the skin lesions based on medical records documenting the numbers of papular AF, fibrous plaque, and hypopigmented macules. We also evaluated the extension of miliary AF and color of AF using photographs. For the two evaluations, we referred to the scorings in the previous study about photograph evaluations for facial AF in TSC (17). Two authors (TO and MO) independently evaluated the photographs, and when the evaluations differed between them, another author (AF) made the final judgement.

We retrospectively reviewed medical records for descriptions of improvements, which were classified as follows: grade (1): no improvement; (2): improvement; (3): marked improvement; or (4): nearly normal skin) (Table 1). Two authors (TO and MO) independently evaluated the improvements, and when the evaluations different between them, another author (AF) made the final judgement.

**Table 1:** Definitions of improvement levels.

	**1: No improvement**	**2: improvement**	**3: Marked improvement**	**4: Nearly normal**
Size of papular angiofibromas	No obvious size reduction	Recognizable reduction^*^ but remaining lesion(s) with <50% volume reduction	Volume reduction (>50%) in all lesions	Flattened (no prominences and smooth skin surface)
Miliary angiofibromas	No obvious improvement	Recognizable flattening and downsizing^*^ but remaining lesion(s) with >20% of beginning extension	Remaining subtle prominences (<20% of beginning extension)	Flattened and smooth (no prominences and smooth skin surface)
Color of angiofibromas	No obvious color change	Recognizable reduction of redness^*^	Remaining subtle redness only in a part of papular lesion(s)	No redness (acceptable remaining post inflammatory hyperpigmentation)
Size of fibrous plaque	No obvious size reduction	Recognizable reduction^*^ but remaining lesion(s) with <50% volume reduction	Volume reduction (>50%) in all lesions	Flattened (no prominences)
Color of hypopigmented macules	No obvious color change	Recognizable reduction of whiteness^*^	Recognizable only when lighted	No white patch

* *Each improvement level was confirmed by physicians and guardians at each visit*.

### Ethics and Informed Consent

The present study was approved by the ethical board of Seirei Hamamatsu General Hospital (Approval No. 2978). Informed consent to use clinical information was obtained from each patient's guardian. We obtained informed consent for the use of photographs from the guardian of Patient #1.

## Results

### Clinical Information and Treatment Adherence

Table 2 describes the clinical information. We collected data for six boys and four girls. One boy (age at treatment initiation: 13.8 years) was eliminated from the study because of poor treatment adherence. The remaining five boys and four girls were included in the analysis. Patients' age at the beginning of the sirolimus gel treatment ranged from 3.5 to 11.0 years (mean: 7.8 years). The follow-up period ranged from 6 to 36 months (14 months). Three patients were followed up for over 24 months. All patients continued the treatments with sirolimus gel until the last follow-up. Two patients (#6 and 9) were treated with oral everolimus for subependymal giant cell astrocytoma (#6 and 9), epilepsy (#6 and 9), and renal angiomyolipoma (#9) before the beginning of the sirolimus gel treatment.

**Table 2:** Clinical information.

**Patient number**	**Age (years) at the beginning of sirolimus gel treatment**	**Sex**	**Gene analysis**	**Facial skin lesions at the beginning of sirolimus gel treatment**	**Treatment of everolimus before sirolimus gel treatment**	**Follow-up period (months)**
1	9.9	Male	NA	Papular AF: 6 Miliary AF: >50%, bilateral Redness of AF: light red Fibrous plaque: 1 Hypopigmented macules: -	–	36
2	8.9	Male	NA	Papular AF: 3 Miliary AF: – Redness of AF: light red Fibrous plaque: 2 Hypopigmented macules: 4	–	24
3	11.0	Female	NA	Papular AF: 6 Miliary AF: >50%, bilateral Redness of AF: red Fibrous plaque: 1 Hypopigmented macules: 1	–	24
4	9.6	Female	*TSC2* mutation	Papular AF: 6 Miliary AF: >50%, bilateral Redness of AF: red Fibrous plaque: 1 Hypopigmented macules: -	–	12
5	6.4	Male	*TSC2* mutation	Papular AF: 2 Miliary AF: >50%, bilateral Redness of AF: light red Fibrous plaque: 1 Hypopigmented macules: –	–	12
6	7.8	Female	*TSC2* mutation	Papular AF: 4 Miliary AF: – Redness of AF: light red Fibrous plaque: – Hypopigmented macules: –	+	12
7	3.5	Female	NA	Papular AF: 2 Miliary AF: >50%, bilateral Redness of AF: light red Fibrous plaque: – Hypopigmented macules: –	–	12
8	9.2	Male	NA	Papular AF: 5 Miliary AF: >50%, bilateral Redness of AF: light red Fibrous plaque: – Hypopigmented macules: –	–	8
9	3.5	Male	NA	Papular AF: 4 Miliary AF: – Redness of AF: light red Fibrous plaque: – Hypopigmented macules: –	+	8

*NA, not analyzed; AF, angiofibromas*.

In Patient #2, AF color worsened because of sun exposure, and sunscreen was applied over the sirolimus gel.

### Skin Lesions Before Sirolimus Gel Treatment

Table 2 also describes the severities of the skin lesions at the beginning of the sirolimus gel treatment. Prior to sirolimus gel treatment, all nine patients presented with 2–6 papular AF on the face. Seven patients (Patients #1, 3–5, 7, 8) presented with diffusely distributed, multiple miliary AF on the cheeks. In the all seven patients, the miliary AF covered >50% of each cheek. The redness of AF was light red in seven patients (Patients #1, 2, 5–9) and red in two patients (#3, 4). Five patients (Patients #1–5) presented with 1–2 fibrous plaques on the forehead. Two patients (Patients #2, 3) presented with one hypopigmented macule on the face. The inter-rater reliability between the two authors (TO and MO) was 1.0.

### Efficacy of Sirolimus Treatment

**Figure 1:**
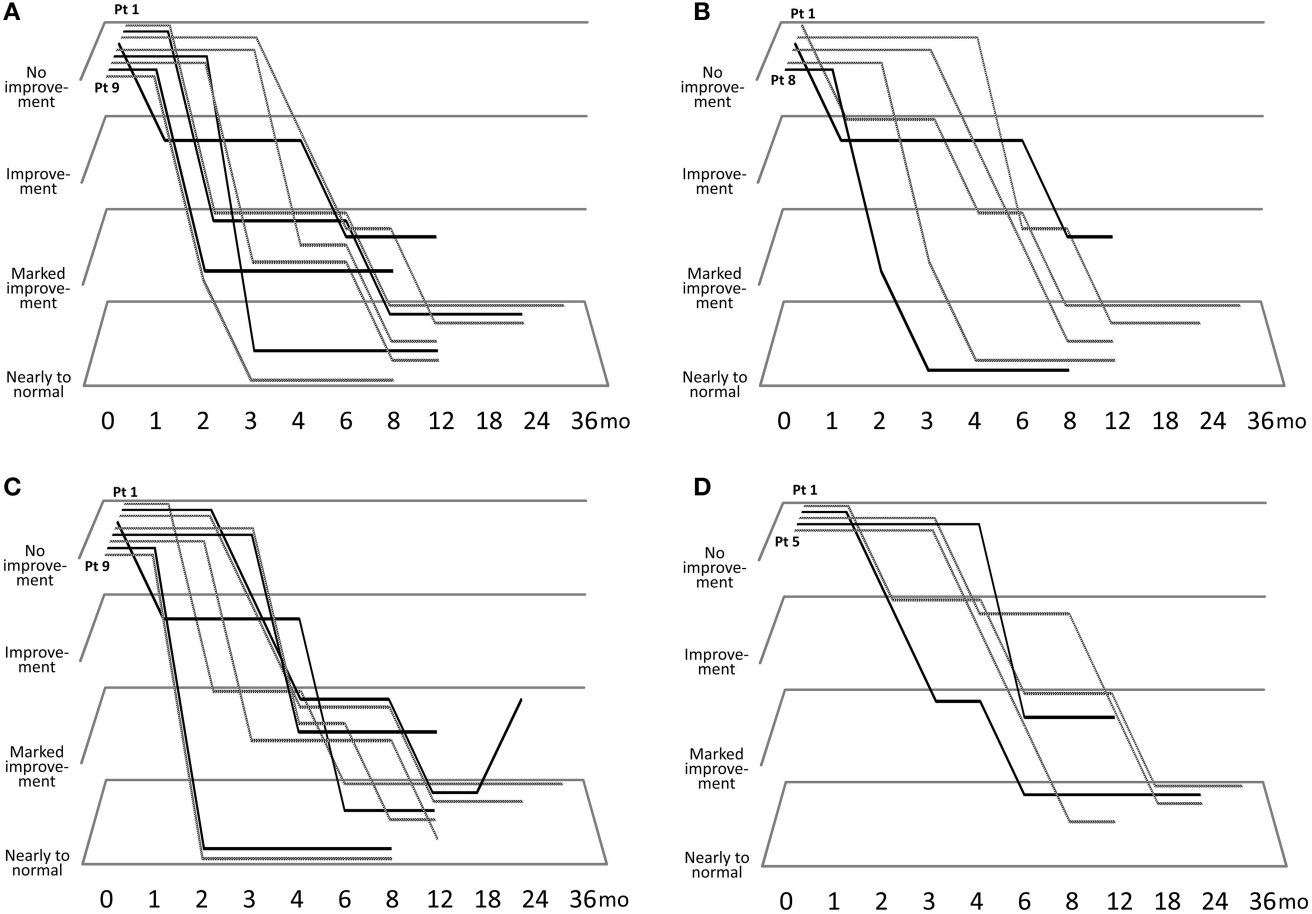
Clinical course of improvements in papular angiofibromas (AF) **(A)**, miliary AF **(B)**, color of AF **(C)**, and fibrous plaque size **(D)**.

**Figure 2:**
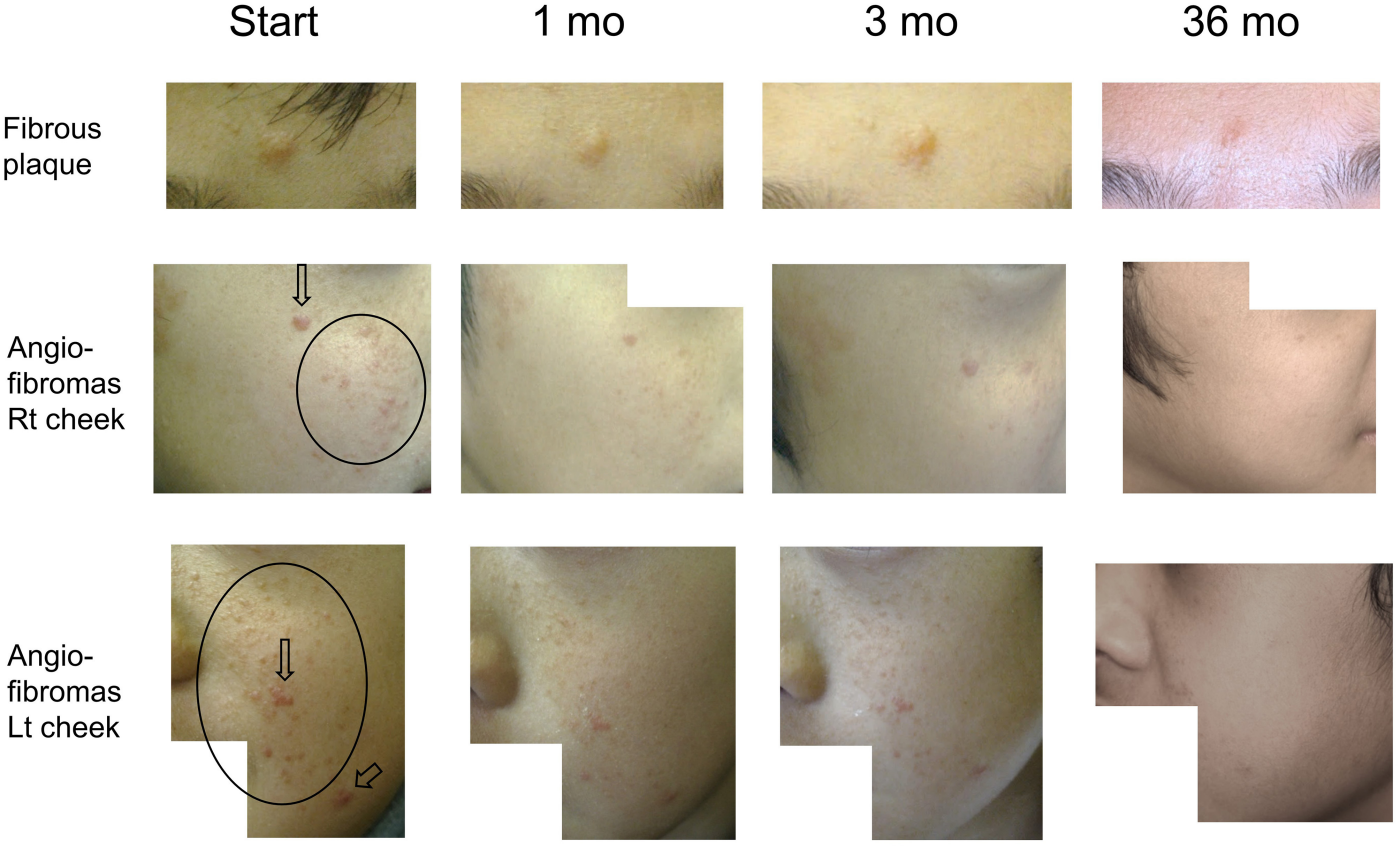
Serial improvements in fibrous plaques (fibrous plaque on forehead [upper line]; angiofibromas [AF] on the right [middle line] and left [bottom line] cheeks) in patient 1. Arrows indicate papular AF. Circles indicate miliary AF. Fibrous plaque sizes are gradually decreased over time, flattening after 36 months of treatment. Papular/miliary AF size and AF redness are also decreased gradually, and the skin exhibits a near-normal appearance until the final follow-up.

Figure 1 describes the efficacy of sirolimus treatment. The Supplementary Table describes the achievement rate of marked improvement or nearly to normal skin at 6 months after the beginning of sirolimus gel treatment. Five patients (Patients #1, 4, 5, 8, 9) with papular AF had persistent post-inflammatory hyperpigmentation at the site of prior AF. Regarding papular AF, miliary AF, redness of AF, and fibrous plaque, the sirolimus gel treatment tended to result in marked improvement or nearly to normal skin within 6 months after beginning the treatment. The median months to achieving these improvement was longer in fibrous plaque (6 months) than in other lesions (3–5 months). The inter-rater reliability between the two authors (TO and MO) was 0.92. The photographs of the improvements in patient 1 were shown in Figure 2.

Two patients (Patients #2, 3) with hypopigmented macules on the face exhibited improvements at 3 and 12 months after the beginning of the treatment, respectively. Despite treatment for 2–3 years, some hypopigmentation remained, and nearly normal skin could not be achieved.

### Side Effects of Sirolimus Treatment

Patient #2 experienced drug acne due to treatment with sirolimus gel. No patients experienced severe skin problems or organ damage during treatment.

## Discussion

In the present study, we investigated the efficacy of early topical sirolimus gel treatment for facial skin lesions in nine children with TSC. All patients exhibited marked improvements in AF color and papular/miliary AF size. Among the three patients followed up for more than 2 years, the skin was nearly normal in appearance, and most improvements were maintained until the final follow-up.

Although some previous studies have investigated the efficacy of topical sirolimus for AF among patients with TSC (7, 8, 10–16), the follow-up period was only 6 months in most of these trials. Thus, the efficacy of long-term sirolimus gel treatment remains to be clarified. In one study with a treatment duration of 8–30 months, crushed sirolimus tablets or sirolimus powder was administered to 19 children (age range: 4–18 years), including 11 children under the age of 12. Excellent efficacy was observed in nine of 19 patients (16). In the present study, we used 0.2% sirolimus gel in 10 children, nine of whom were under the age of 10. The gel formulation is better at penetrating the skin than ointments or other vehicles (10), leading to near-complete resolution of AF in all three children treated for more than 1 year.

In this study, we examined whether treatment initiation during the early stages of AF development can improve skin condition to near-normal levels. Sirolimus efficacy for AF was generally observed after 3–6 months of treatment. The skin surface was smooth and nearly normal in the three patients treated for more than 2 years. This finding suggests that early initiation of sirolimus treatment can lead to near-normal skin in the long term, possibly for the life span, in patients with TSC exhibiting AF.

Previous research has indicated that the response rate to sirolimus gel is lower for fibrous plaques than for AF (18). In the present study, decreases in fibrous plaque size were more gradual than those in AF size. Nonetheless, three of five patients achieved improvements after 6–18 months of treatment. Long-term continuous treatment may reduce plaque size despite its high collagen content (19). In contrast, the efficacy of sirolimus gel for hypopigmented macules was limited. Although improvements in hypopigmentation were observed 3–8 months after treatment, nearly normal skin could not be achieved. However, these evaluations were performed in only two patients. Further studies are required to verify the efficacy of topical sirolimus for hypopigmented macules.

The efficacy of topical sirolimus is thought to achieve a plateau in patients with relatively advanced AF (14, 16). Lee et al. investigated the efficacy of sirolimus ointment in 36 patients with AF, including three patients treated for over 1 year. The authors observed no further dramatic improvement after 20 weeks of treatment (14). Although the efficacy of sirolimus gel for AF is greater than that for topical sirolimus using other vehicles (8, 11), sirolimus gel may exhibit limited efficacy for enlarged AF, similar to everolimus, which cannot diminish SEGA completely. Early initiation of sirolimus gel treatment prior to lesion growth may help to maintain a near-normal skin condition in patients with TSC. However, our study is limited by the small number of patients with long-term follow-up. Future studies with more patients and a longer follow-up are necessary.

In conclusion, the present study demonstrated the efficacy of sirolimus gel for skin lesions including AF, fibrous plaques, and hypopigmented macules in children with TSC. After several months of treatment, children exhibited near-normal skin, which was maintained for several years. Thus, early intervention with sirolimus gel is recommended for children with TSC.

## Data Availability Statement

De-identified data related to the current study are available on request to any qualified researcher after certification by the ethical board of Seirei Hamamatsu General Hospital, until 3 years after publication.

## Ethics Statement

The studies involving human participants were reviewed and approved by the ethical board of Seirei Hamamatsu General Hospital. Written informed consent to participate in this study was provided by the participants' legal guardian/next of kin. Written informed consent was obtained from the individual(s), and minor(s)' legal guardian/next of kin, for the publication of any potentially identifiable images, or data included in this article.

## Author Contributions

TO contributed to conceptualizing, drafting, and revising the study, analyzing, acquiring, and evaluating the skin lesions clinically, and interpreting the data. AF contributed to conceptualizing and revising the study and reviewing the photographs. MO contributed to conceptualizing and revising the study and evaluating the skin lesions clinically. HE contributed to conceptualizing and revising the study.

### Conflict of Interest

The authors declare that the research was conducted in the absence of any commercial or financial relationships that could be construed as a potential conflict of interest.
